# Therapeutic Options for the Treatment of Darier’s Disease: A Comprehensive Review of the Literature

**DOI:** 10.1177/12034754211058405

**Published:** 2021-11-28

**Authors:** Nardin Hanna, Megan Lam, Patrick Fleming, Charles W. Lynde

**Affiliations:** 1 6363 Faculty of Medicine, University of Ottawa, ON, Canada; 2 12362 Faculty of Medicine, McMaster University, Hamilton, ON, Canada; 3 7938 Division of Dermatology, University of Toronto, ON, Canada; 4 Lynde Institute of Dermatology, Markham, ON, Canada

**Keywords:** Darier’s disease, treatment, genodermatosis

## Abstract

Darier’s disease (also known as keratosis follicularis or dyskeratosis follicularis) is an autosomal dominant inherited disorder which manifests as hyperkeratotic greasy papules in the first or second decade of life. Aside from symptom management and behavioral modifications to avoid triggers, there are currently no validated treatments for Darier’s disease (DD). However, a variety of treatments have been proposed in the literature including retinoids, steroids, vitamin D analogs, photodynamic therapy, and surgical excision. The purpose of this review article is to identify therapeutic options for treating DD and to outline the evidence underlying these interventions. A search was conducted in Medline for English language articles from inception to July 4, 2020. Our search identified a total of 474 nonduplicate studies, which were screened by title and abstract. Of these, 155 full text articles were screened against inclusion/exclusion criteria, and 113 studies were included in our review. We identified Grade B evidence for the following treatments of DD: oral acitretin, oral isotretinoin, systemic Vitamin A, topical tretinoin, topical isotretinoin, topical adapalene gel, topical 5-flououracil, topical calciptriol and tacalcitol (with sunscreen), grenz ray radiation, and x-ray radiation. All other evidence for treatments of DD consisted of case reports or case series, which is considered grade C evidence. Considering the quality and quantity of evidence, clinicians may consider initiating a trial of select topical or oral retinoids first in patients with localized or generalized DD, respectively.

## Introduction

Darier’s disease (also known as keratosis follicularis or dyskeratosis follicularis) is an autosomal dominant inherited disorder with variable expressivity.^1,2^ It occurs through mutations in the ATP2A2 gene, which encodes the sarcoplasmic reticulum ATPase type two (SERCA2) calcium pump.^1^ This mutation manifests as hyperkeratotic greasy papules in the first or second decade of life.^1^ The papules predominantly erupt on the seborrheic surfaces of the skin and are frequently accompanied by scaling and scabbing.^3^ On histology, Darier’s disease (DD) can be characterized by its distinctive features which include: acantholysis resulting in the formation of suprabasal clefts, and dyskeratosis involving corps ronds and grains.^3^ The prevalence of DD is estimated to be between one in 30 000, and one in 100, 000.^1^

There are currently no validated treatments for DD, with avoidance of triggers and symptom management being the mainstay.^3^ However, a variety of treatments have been proposed in the literature including retinoids, steroids, vitamin D analogs, photodynamic therapy and surgical excision.^2^ As such, identifying a targeted treatment for DD is a primary concern and was listed as a priority translational dermatology research question by over 70% of participants in the UK eDelphi study group.^4^ The purpose of this review article is to identify the therapeutic options for treating DD and to outline the evidence underlying these interventions. Identifying potential treatments and grading the evidence underlying these therapies can help create a therapeutic algorithm for treating DD patients. In addition, a review of effective treatments can offer potential treatment options for recalcitrant cases of the disease. Finally, this review may also highlight encouraging avenues for future research trials of interventions for DD.

## Materials and Methods

The study was performed according to the Preferred Reporting Items for Systematic Reviews and Meta-analyses (PRISMA) guidelines, and registered in PROSPERO (International Prospective Register of Systematic Reviews, registration number CRD42020205964).^5^ A search was conducted in Medline for English language articles from database inception to July 4, 2020. The search strategy consisted of keywords and subject headings relating to the following two concepts combined with Boolean operators: Darier’s disease, and treatments. All search results were uploaded into Covidence Software for independent title/abstract and full-text screening by two reviewers (N.H. and M.L.). Articles were included if they provided data on treatments of DD and their outcome. Treatments were defined as interventions which had any positive effect in improving DD. We excluded articles which investigated the symptomatic treatment of patients with DD or which provided information on the use of etretinate in the treatment for DD, as this drug has been discontinued in North America. We also excluded review articles, conference abstracts and studies which were not available in the English language or studies for which the full text was not available. Disagreements were resolved through discussion between the reviewers.

### Quality Assessment

We used a modified Newcastle-Ottawa Scale to assess the quality of included case reports, case series, and uncontrolled clinical trials or non-comparative cohort studies.^6^ We used the Cochrane Risk Of Bias In Non-Randomized Studies of Interventions (ROBINS-I) tool to assess the quality of controlled non-randomized trials. Two reviewers (N.H. and M.L.) assessed the quality of each included study. Disagreements were resolved through discussion between the reviewers.

### Data Extraction and Synthesis

Data extraction was performed by the consensus of two independent reviewers (N.H. and O.S.) using a standard abstraction form including information about author, year of publication, study type, therapeutic modality used, regimen, duration of treatment, special indications, associated side effects or adverse events, and outcome of treatment. A series of evidence tables containing the extracted information were created. Evidence will be graded according to the Levels of Evidence to classify the quality of evidence support each intervention as follows: grade A, systematic reviews and meta-analysis or randomized control trials with consistent findings; grade B, systematic reviews/meta-analysis of lower-quality studies or studies with inconsistent findings, lower-quality clinical trials, cohort studies and case-control studies; Grade C, consensus guidelines, usual practice, expert opinion, case series and case reports.^7^ Grade A, B, and C quality of evidence will correspond to Grade one reccomendation (strong recommendation; high-quality evidence), two A reccomendation (weak recommendation; limited quality, patient-oriented evidence), and two B reccomendation (weak recommendation; low-quality evidence) strength of recommendation, respectively.^7^

## Results

Our search identified a total of 474 nonduplicate studies, which were screened by title and abstract. Of these, 155 full text articles were screened against inclusion/exclusion criteria, and 113 studies were included in our review (Supplemental Figure 1). Risk of bias assessments for included studies can be found in Supplemental Tables 1 and 2. Therapies used for the treatment of DD can be subdivided into four categories: oral retinoids, other oral therapies, topical, and procedural (Supplemental Tables 3-6). Combination therapies reported in the literature for the treatment of DD are listed in Supplemental Table 3.

### Systemic Therapies

#### Oral retinoids

We identified Grade C evidence for the use of oral retinoids as a treatment for DD when they were evaluated aggregately, likely through their antiproliferative effects on keratinocytes.^8,9^ In two included cohort studies with a total of 118 patients, 108 patients identified a benefit, however, significant side effects were reported in the majority of patients with this therapy, resulting in discontinuation by a significant number.^8,9^ Of note, oral retinoids used in the included studies include or may include etretinate and as such, results may not reflect the true efficacy of oral retinoids available in the United States and Canada at this moment.^9^

#### Acitretin

There was Grade B evidence for the use of the oral retinoid, acitretin in improving DD^10-23^. In two clinical trials evaluating the use of acitretin in 14 patients, all patients experienced improvement, with one study reporting significant improvement or complete clearance in four of five patients. There were significant side effects reported with the use of acitretin including: aridity cheilitis, increasing fragility of skin, dry mouth and skin, anorexia, pruritus, dry eye, hyperorexia, hearing handicap, brittle nail in one finger, acral hemorrhagic lesions, and mild elevation of serum triglyceride levels.^10,18,21,23^ Additionally, long-term treatment with acitretin may be necessary, as reoccurrence of DD has been noted in patients who terminate treatment.^12,18^

#### Isotretinoin

There was also Grade B evidence for the use of the oral retinoid, isotretinoin in treating DD^24-33^. In trial by Dicken et al., 98 of 104 patients with DD who used isotretinoin experienced improvement of their symptoms after 16 weeks of treatment.^28^ Patients whose disease persisted after this first round of therapy, received further treatment rounds, with similarly high rates of success.^28^ Three other trials with a cumulative 17 patients reported moderate to significant clearance in 14 patients, and mild improvement in 3 patients^24,25,29^.^25^ Significant side effects reported with the use of oral isotretinoin including: epistaxis, cheilitis, xerosis, skeletal hyperostosis (after 6 to 12 months of use), conjunctivitis, facial dermatitis, rhinitis sicca with nosebleed, skin fragility, itching, headache, appetite changes, fingertip peeling, inflammation of the urethral meatus, hair thinning, dryness of mouth (with feeling of thirst), allergic reaction, and elevated AST, ALT, ALP, and ESR^24-27^. In addition, termination of oral isotretinoin has resulted in relapse of DD.^26-29,31,33^ As such, it may be necessary to take isotretinoin continuously on maintenance dosing.

#### Alitretinoin

There were several case reports which (Grade C) reported on the benefits of alitretinoin, another oral retinoid^34-37^. All patients using oral alitretinoin were reported to experience moderate to significant improvement of their DD^34-37^. The side effects of alitretinoin include mild eye dryness, moderate reversible hair loss, headache, elevated total cholesterol, and low-density lipoprotein levels.^36,37^ Most notably, alitretinoin was used in women of child-bearing age, due to its shorter half-life, and correspondingly lower risk of teratogenicity when compared to other oral retinoids.^34^ However, similar to other oral retinoids, DD has been reported to relapse after discontinuation or decreased frequency of this treatment, and as a result, continuous treatment may be required^34-36^.

#### Vitamin A Analogs

A variety of other systemic Vitamin A medications have been reported to treat DD including: Vitamin A, Vitamin A acid (tretinoin), and Vitamin A palmitate^38-42^. The active metabolite of Vitamin A medications is retinoic acid, which similar to other retinoids, regulates hyperproliferation of keratinocytes.^40^ Our literature search identified Grade B evidence reporting partial to significant clinical improvement in patients receiving systemic Vitamin A^39,41,42^. In a trial of seven patients receiving vitamin A, six experienced significant clinical improvement of their DD, while one patient experienced no clinical response.^42^ However, side effects of mild desquamation, pruritis, dry/scaling lips, dry nose, dry skin, headaches, sleepiness, and drowsiness were experienced by patients on this therapy^39^ . There was also evidence of partial relapse of DD after completing treatment.^39^ The use of oral Vitamin A Acid (tretinoin) was reported by one case series consisting of 8 patients (Grade C).^38^ All patients were able to control their DD using tretinoin, however side effects of cheilitis, rhinitis sicca, and skin cleavage, were observed.^38^ Additionally, relapses of DD was reported with the discontinuation of this medication.^38^ Finally, there was one case report describing the use of Vitamin A palmitate in the treatment of DD (Grade C).^40^ The patient experienced significant clinical improvement which persisted with the use of maintenance therapy, without any reported side effects.^40^

#### Systemic Immunomodulators

There was Grade C evidence for the use of methylprednisone, immunoglobulin, and cyclosporine as treatments for DD^43-47^. These medications inhibit the inflammatory cycle, which may have a role in the exacerbation of DD^47-50^. Two case reports investigated the use of methylprednisone and intravenous immunoglobulin and reported complete remission and significant improvement of DD, respectively. There were also three case reports with a total of four patients which described the benefits of cyclosporine in treating DD^45-47^. Patients using cyclosporine reported adverse events (significant hypertension and acute renal failure) after starting the medication, and as a result, treatment was terminated.^45,47^ Similar to other treatments, it was also reported that DD may relapse after discontinuation of cyclosporine.^47^

#### Oral Magnesium

There was one case report of oral magnesium used to successfully treat DD, without evidence of relapse for 1 month after treatment (Grade C).^51^ Due to the role of magnesium in inhibiting calcium efflux from cells, it may benefit patients with SERCA2 dysfunction (as in DD).^51^ However, as reported in the article, it is difficult to ascertain whether oral magnesium can treat DD or whether positive results are due to the clinical course of DD, which may often undergo remission spontaneously.^51^

#### Systemic Penicillamine

The proposed mechanism of action of penicillamine involves its role in chelating copper used in the disulphide bonds of keratin, thereby decreasing keratinocyte proliferation.^52^ There was some evidence to support a trial of penicillamine as a treatment for DD, although it was poor and inconsistent (Grade C).^52^ In a case series of six patients receiving this medication, three patients experienced moderate to significant improvement of their DD, while three patients experienced only minimal or negligible improvement of their DD.^52^ Urticaria and eczema was also reported as a side effect of this treatment regimen in three of the six patients.^52^ Relapse of DD was also reported in every patient after discontinuation of penicillamine, suggesting that a long-term regimen or multiple treatment courses may be required.^52^

#### Systemic Antibiotics

Although antibiotics are commonly used by patients with DD to treat superimposed cutaneous infections, we identified multiple case reports which investigated the use of doxycycline for the clearance of DD (Grade C).^53,54^ Doxycycline belongs to the class of tetracyclines which can suppress inflammation, epidermal proliferation, collagenase activity and infectious exacerbations, all of which are mechanisms of DD.^55^ In the two patients treated with doxycycline, there was significant clinical improvement to complete remission of DD, which persisted for 3 months with maintenance therapy at the time of the report.^53,54^

#### Oral Contraceptives

There have been multiple reports of exacerbations of DD associated with menstruation or pregnancy.^56^ Correspondingly, we found Grade C evidence of the use of estrogen as a treatment for DD in female patients experiencing flares of their skin disease when menstruating.^56^ Oostenhrink et al. reported moderate clinical improvement with the use of Microgynon 50 (combination estrogen/progesterone pill) in one patient who experienced flares of her DD during menstruation.^56^

### Topical Therapies

#### Topical Tretinoin

Although the use of tretinoin (also known as all-trans-retinoic acid, retinoic acid or vitamin A acid) was the most frequently reported topical treatment for DD, the evidence for its efficacy was contradictory and of limited quality (Grade C).^8,12,19,57-64^ In Goh’s retrospective study of eight patients treated with topical retinoic acid, only two experienced mild benefit from this treatment.^12^ In one controlled trial of one patient with DD, areas treated with topical all-trans-retinoic acid were moderately improved, meanwhile placebo-treated areas were unchanged.^64^ In 12 alternate cases, however, therapy with tretinoin resulted in partial to complete remission of DD.^19,57-64^ Among patients, side effects of mild itching and tenderness, inflammation and desquamation were reported.^57,58^ Therapy with all-trans-retinoic acid was ultimately discontinued in two patients in Goh’s study, and the single patient in the control trial due to side effects of severe irritation, erythema and burning.^12,64^ In Burge et al’s retrospective non-comparative cohort study of 163 patients with DD, it was reported that all patients who had been prescribed topical retinoic acid eventually discontinued treatment as it had a tendency to irritate skin.^65^ There was also evidence of relapse after discontinuation of treatment with topical tretinoin.^57^

#### Topical Isotretinoin

Topical isotretinoin (also known as 13-cis-retinoic acid) was also identified as a treatment for DD in the literature, with variable effects on patients ranging from no response to complete remission of involved skin (Grade B evidence).^64-67^ In one trial of topical isotretinoin used to treat patients with DD, six of twelve patients achieved partial or complete remission of their DD.^62^ However, when visual analog scale ratings were compared before and after 3 months of treatment, there was no significant difference between the two ratings (mean difference 12.2 mm, SD = 31.4, *P* = .279).^62^ There were also several side effects reported with the use of topical isotretinoin in the literature. These include mild-severe burning, erythema, irritation, itching and tenderness.^64,65,67^

#### Topical Adapalene

Adapalene was another retinoid identified in our search which was able to provide moderate to significant clinical improvement of DD (Grade B evidence).^68-70^ There were no notable side effects noticed on this treatment, however reoccurrence was reported after treatment was stopped^68-70^.

#### Topical Tazarotene

In two case reports identified in our search, Tazarotene (a topical retinoid) resulted in complete remission of DD (Grade B).^71,72^ There were no notable side effects or reoccurrence reported for up to 24 months after remission.^71,72^

#### Topical Synthetic Vitamin D Analogs

Vitamin D encourages differentiation and inhibits proliferation of epidermal keratinocytes, making it a potential therapeutic agent for DD.^73^ Correspondingly, there was grade B evidence supporting the efficacy of Vitamin D analogs Calcipotriol and Tacalcitol as treatments for DD in the literature.^69,73,74^ In a randomized right/left control trial of eleven DD patients, Calciptriol resulted in worsening DD and lesional-perilesional skin irritation in eight patients (with seven patients out of the recruited twelve dropping out due to adverse events), mild improvement in two patients, and moderate improvement in one patient.^72^ On the placebo side, however, only five of eleven patients experienced worsening DD, while two experienced moderate improvement and four experienced no changes.^72^ Alternately, Tacalcitol (used with sunscreen) was not found to have any notable side effects, however outcomes were variable, ranging from negligible response to significant clinical improvement of DD lesions.^69,74^

#### Topical Fluocinonide

There was Grade C evidence for the use of Fluocinonide, a corticosteroid, as a treatment for DD in the literature.^75^ The use of topical Fluocinonide was evaluated in one case report and resulted in moderate to significant clinical improvement of DD in one patient.^75^

#### Topical Calcineurin Inhibitors

As their name suggests, calcineurin inhibitors bind calcineurin, diminish T-cell proliferation, thereby inhibiting inflammation involved in symptomatic DD.^76^ Two types of topical calcineurin inhibitors were identified as treatments of DD in the literature: tacrolimus and pimecrolimus.^76,77^ Tacrolimus was described in one case report and resulted in the complete remission of DD lesions in one patient (Grade C evidence).^77^ With the use of maintenance therapy, this remission lasted for at least 12 months of follow-up.^77^ Pimecrolimus also resulted in the complete remission of DD lesions in one patient without any notable side effects (Grade C evidence).^76^

#### Topical Non-steroidal Anti-inflammatories

The use of diclofenac sodium gel for treatment of DD was described in multiple reports in the literature (Grade C evidence).^78-80^ The proposed mechanism of action for this treatment is through COX-2 inhibition, which has the effect of upregulating production of the SERCA2 protein.^78^ Patients using this treatment experienced moderate to significant clinical improvement of their DD lesions, without any notable side effects. However, there was evidence of relapse after discontinuation of diclofenac sodium treatment.^79^

#### Topical 5-Fluouracil

There was Grade B evidence for the use of the chemotherapeutic agent, 5-flououracil, for the treatment of DD^81-83^. The mechanism of action of this therapy is inhibition of DNA synthesis, which decreases the hyperproliferation seen in DD.^82^ Variable success was reported with the use of this therapy ranging from no clinical response to complete remission^81-83^. In one non-controlled trial of four patients with DD, two patients experienced significant improvement of their DD, as well as symptomatic relief of symptoms including itching and burning.^83^ However, in the two patients who experienced treatment response, post-inflammatory hyperpigmentation was reported.^83^ Remission periods of two to six months were reported with the use of 5-fluouracil, with evidence of relapse in some patients.^82,83^

### Procedural Therapies

#### Surgical Excision and Dermabrasion

Surgical excision was used to treat DD in several case reports with outcomes ranging from significant clinical improvement to complete remission (Grade C evidence).^11,84-90^ In these cases, partial-thickness and full-thickness surgical excision was used to remove areas of involved skin (or nails) with reconstruction using rotation flaps or skin grafts, when necessary.^11,84-90^ In one case, bilateral reduction mammaplasty was also used to achieve significant improvement of inframammary DD.^89^ Reported complications with surgical excision of DD include necrosis and exudate from wound, necrosis of the nipple-areolar complex (in the case of mammaplasty), wound dehiscence, infection, scar formation and hypopigmentation.^84,86,87,89^ Remission was maintained in some reports for as long as 3 years in skin excisions and 7 years in nail excisions.^11,90^ However, there was also evidence of relapse of DD in areas where surgical excision was less deep.^85,90^

There was Grade C evidence for use of dermabrasion for the treatment of DD.^91^ Patients experienced remission of at least 75% of the skin which was treated with dermabrasion for at least two and a half years.^91^ Similar to other studies, surgical intervention was successful when the papillary dermis was treated in addition to the superficial epidermal layers.^91^

#### Lasers

Lasers are believed to have their effect by destroying the superficial layer of skin (up to papillary dermis), and the eccrine glands, which have a role in exacerbating DD.^92,93^ The use of a carbon dioxide laser to treat DD was reported in several patients and resulted in outcomes ranging from moderate clinical improvement to complete remission (Grade C evidence).^94-98^ Side effects of this therapy included: irritation, edema, and erythema (may be transient or prolonged).^94,96,97^ Although remission durations of up to 9 years were reported after carbon dioxide laser therapy, reoccurrence of DD lesions was noted in multiple patients (although it was less severe).^95,96,98^

Erbium-doped yttrium and Erbium-doped fiber laser treatment was reported by Grade C evidence.^99,100^ Outcomes of patients treated with this therapy ranged from experiencing significant clinical improvement of their DD lesions to complete remission, with remissions lasting up to eighteen months at time of reporting.^99,100^ There were several side effects reported, however, including hypopigmentation (atrophic hypopigmented spots), pain, and transient erythema and edema.^99,100^

Flashlamp-pumped pulsed-dye lasers were reported on multiple occasions as significant potential in treating DD (Grade C evidence).^101,102^ In one case series of eight patients who received therapy with flashlamp-pumped pulsed-dye laser, all patients experienced significant clinical improvement or complete remission of their DD 3 months after treatment, with remission lasting up to 18 months in six patients.^98^ Side effects reported with this treatment include: purpura with mild crusting, and flare of herpes-simplex virus infection (occurred in two of eight patients in case series).^101,102^ There was also one case of DD treated with near-infrared 1450 nm wavelength diode laser, which resulted in complete remission of DD (Grade C).^95^ Remission was reported for 3 years at the time of report, without any notable side effects.^95^

#### Radiation

Although the exact mechanism of action is unknown, a variety of radiation techniques have been trialed with varying success in treating DD including Grenz Ray, electron beam radiation, conventional x-rays, and radiotherapy.^103^ Grenz ray radiation was compared with conventional x-ray and no radiation therapy in a single patient controlled trail(Grade B evidence). Outcomes were not significantly different after treatment completion, with improvement in all treatment areas.^104^ As such, it is difficult to ascertain whether improvement of DD can be attributed to these radiation therapies, or whether improvement occurred due to the spontaneous trajectory of DD.^104^

There was Grade C evidence for the use of electron beam radiation therapy as a treatment for DD.^103,105,106^ Outcomes of significant clinical improvement to complete remission of DD were reported in patients who were treated with localized and total electron beam therapy.^105,106^ Side effects observed after localized electron beam therapy included temporary local dermatitis and moist desquamation, striae in treatment areas, and mild disease flare outside the treatment area.^105^ However, after total electron beam radiation, more severe side effects were observed including severe dermatitis, skin pain, nausea, and vomiting.^105^ Ulceration and sclerosis also developed in areas treated with electron beam radiation and persisted after the treatment course was complete.^106^ These adverse events resulted in prolonged hospitalization of one patient and admission to the intensive care unit.^105^

Alternately, photon radiation therapy was chosen to treat DD patients with thickened hyperkeratotic contours due to its ability to penetrate more deeply.^106^ Unlike electron beam radiation therapy, there were no significant side effects reported with the use of this treatment, except for temporary pain.^106^ Patients in this case series experienced moderate to significant improvement of their DD after one to two courses of photon radiation therapy (Grade C evidence).^106^

There was Grade C evidence for the use of radiotherapy as a treatment for DD.^107,108^ In multiple case reports, patients who were treated with radiotherapy for a malignancy experienced complete remission of their DD, with only mild side effects of local dermatitis or temporary exacerbation of their DD lesions.^107,108^ However, it is important to consider that has also been evidence to suggest that radiotherapy may trigger the onset of DD.^109^

#### Photodynamic Therapy

Photodynamic therapy uses UV radiation to cause reactive oxygen species formation and subsequently apoptosis in involved skin.^110^ There is varying evidence for the use of photodynamic therapy in the treatment of DD with some literature reporting complete remission, and others reporting only temporary clearing followed by exacerbation (Grade B).^110-112^ In some patients, remission was achieved for a duration of 7 months to 3 years at time of report.^110^

## Discussion

**Figure 1:**
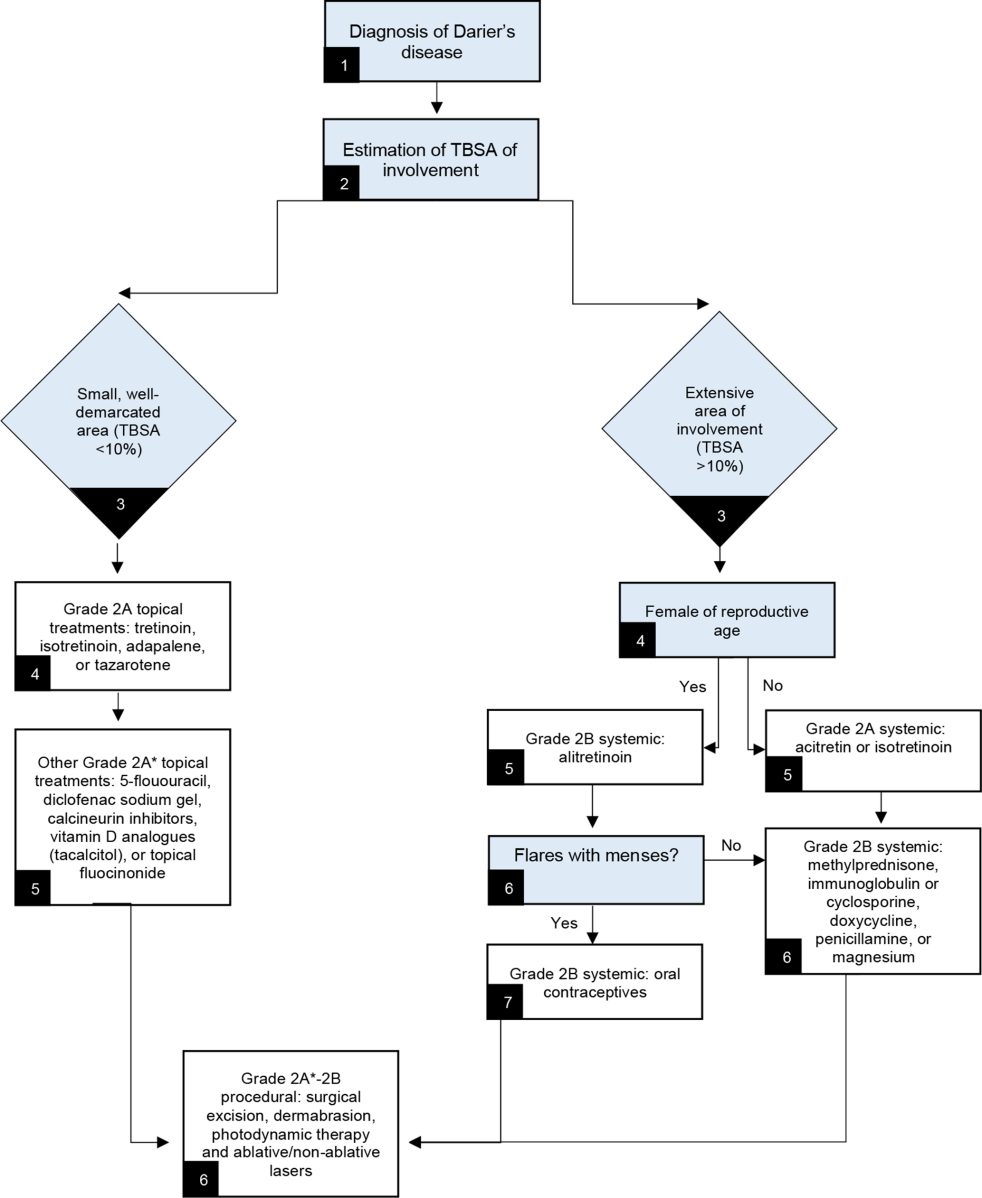
Recommended treatment algorithm for management of Darier’s disease. TBSA: Total Body Surface Area . *Grade 2A recommendation with smaller quantity of evidence.

The following treatments for DD were identified as having the strongest quality of evidence: oral acitretin, oral isotretinoin, systemic Vitamin A, topical tretinoin, topical isotretinoin, topical adapalene gel, topical 5-flououracil, topical calciptriol and tacalcitol (with sunscreen), grenz ray, and x-ray. The evidence underlying each of these treatments was awarded a grade B which corresponds to a 2A grade of recommendation for each treatment (weak recommendation due to limited quality patient-oriented evidence). However, despite the similar quality of evidence scores, there were significantly more studies highlighting the use of oral acitretin and isotretinoin and topical tretinoin and isotretinoin as effective treatments for DD (although response was variable from negligible to complete remission). All other evidence for treatments of DD were in the form of case reports or case series, which are considered grade C evidence. Based on this data, a recommended algorithm was created for managing DD (Figure 1). This summary does not include data about effective combination treatments used for managing DD. In general, most combination treatments consisted of 2 single agents from the options presented in this review. One except was naltrexone which was reported excusively in combination with magnesium and other oral retinoids.^113^ A summary of combination treatments for DD can be found in Supplemental Table 7.

One consideration when choosing a treatment for DD is the risk of relapse with these therapies. In almost all trialed interventions, relapse was reported in subjects after discontinuation. As such, intermittent courses of therapy or long-term maintenance dosing may be required to prevent the recurrence of this disease. However, there were no reports of relapse in patients trialed on oral magnesium, topical isotretininoin, topical tazarotene, topical calciptriol, topical talcalcitol, erbium-doped yttrium radiation, grenz ray radiation, x-ray radiation, electron beam radiation, photon radiation, and radiotherapy. It is difficult to ascertain whether patients trialed on these therapies did not experience relapse at any point after treatment as time of follow-up was either not reported in many studies or was very short (1 month to 3 years). Long-term studies investigating the use of these interventions will be required to make recommendations on treatments providing the longest remission periods. As it stands the treatment with longest remission period which was recorded in the literature was through surgical excision which lasted up to 9 years in one patient at time of report.

### Limitations

Our study had several limitations. First, most articles identified in our search were case reports or case series, which are limited by reporting bias. Due to the low prevalence of DD, clinical trials or cohort studies also had small sample sizes, which limits the power of the results. Future studies may consider high-powered trials of DD treatments with the goal of providing grade A evidence for treatments. Finally, there is no objective measure of DD control, and as such, most studies used subjective clinician ratings of response to treatments. This may have resulted in inter-observer disparities when evaluating response.

## Conclusion

DD is an autosomal dominant genodermatosis which presents with hyperkeratotic papules that may be accompanied by scaling and crusting.^2^ A variety of treatments which have been used to treat this disease in the literature, although the quality of the evidence remains poor (consisting predominantly of case reports and non-experimental studies). The best quality and quantity of evidence exists for the use of select oral and topical retinoids. Emerging evidence suggest interventional treatments such as surgical excision, lasers, and radiotherapy, may be effective in the treatment of DD. Further studies on efficacy and safety in comparative randomized trials would be important.

## Supplemental Material

Supplementary Material 1 - Supplemental material for Therapeutic Options for the Treatment of Darier’s Disease: A Comprehensive Review of the Literature Click here for additional data file. Supplemental material, Supplementary Material 1, for Therapeutic Options for the Treatment of Darier’s Disease: A Comprehensive Review of the Literature by Nardin Hanna, Megan Lam, Patrick Fleming and Charles W. Lynde in Journal of Cutaneous Medicine & Surgery

Online supplementary file 1 - Supplemental material for Therapeutic Options for the Treatment of Darier’s Disease: A Comprehensive Review of the Literature Click here for additional data file. Supplemental material, Online supplementary file 1, for Therapeutic Options for the Treatment of Darier’s Disease: A Comprehensive Review of the Literature by Nardin Hanna, Megan Lam, Patrick Fleming and Charles W. Lynde in Journal of Cutaneous Medicine & Surgery
