# Supplementary material for: Therapeutic Options for the Treatment of Darier’s Disease: A Comprehensive Review of the Literature
Source: J Cutan Med Surg. 2021 Nov 28;26(3):280–90. doi: 10.1177/12034754211058405 (PMC9125141; doi:10.1177/12034754211058405)
Supplement: Supplementary Material 1 - Supplemental material for Therapeutic Options for the Treatment of Darier’s Disease: A Comprehensive Review of the Literature [file sj-docx-1-cms-10.1177_12034754211058405.docx]

**Supplemental Material**

**Therapeutic options for the treatment of Darier’s disease: A Comprehensive Review of the Literature**

N Hanna^1^, BHSc, M Lam^2^, BSc, P Fleming^3^, MD, C Lynde^3,4^, MD

1. University of Ottawa, Faculty of Medicine, Ottawa ON, Canada
2. McMaster University, Faculty of Medicine, Hamilton ON, Canada
3. Division of Dermatology, University of Toronto, Toronto ON, Canada
4. Lynde Institute of Dermatology, Markham ON, Canada

**Supplementary Table 1: Newcastle-Ottawa Scale for case reports, case series, and non-controlled clinical trials/noncomparative cohort studies**

| Study | Selection | Ascertainment | | Causality | | | | Reporting | Total Score out of 8 |
| --- | --- | --- | --- | --- | --- | --- | --- | --- | --- |
|  | Does the patient(s) represent(s) the whole experience of the investigator (centre) or is the selection method unclear to the extent that other patients with similar presentation may not have been reported? | Was the exposure adequately ascertained? | Was the outcome adequately ascertained? | Were other alternative causes that may explain the observation ruled out? | Was there a challenge/rechallenge phenomenon? | Was there a dose–response effect? In procedural cases, was temporality established? | Was follow-up long enough for outcomes to occur? >/=2mo? | Is the case(s) described with sufficient details to allow other investigators to replicate the research or to allow practitioners make inferences related to their own practice? |  |
| Abe 2010 |  |  |  |  |  |  |  |  | 4 |
| Ahcan 2009 |  |  |  |  |  |  |  |  | 5 |
| Amerio 2007 |  |  |  |  |  |  |  |  | 6 |
| Anuset 2014 |  |  |  |  |  |  |  |  | 7 |
| Archer 1989 |  |  |  |  |  |  |  |  | 4 |
| Ashok Kumar 2020 |  |  |  |  |  |  |  |  | 4 |
| Avery 2010 |  |  |  |  |  |  |  |  | 5 |
| Ayres 1983 |  |  |  |  |  |  |  |  | 7 |
| Baran 2005 |  |  |  |  |  |  |  |  | 4 |
| Beer 1966 |  |  |  |  |  |  |  |  | 7 |
| Beier 1999 |  |  |  |  |  |  |  |  | 7 |
| Beiu 2019 |  |  |  |  |  |  |  |  | 4 |
| Benmously 2015 |  |  |  |  |  |  |  |  | 5 |
| Bhat 2010 |  |  |  |  |  |  |  |  | 3 |
| Blanchet-Bardon 1991 |  |  |  |  |  |  |  |  | 4 |
| Boehmer 2019 |  |  |  |  |  |  |  |  | 5 |
| Borgogna 2009 |  |  |  |  |  |  |  |  | 5 |
| Brown 2010 |  |  |  |  |  |  |  |  | 7 |
| Burge 1992 |  |  |  |  |  |  |  |  | 2 |
| Burge 1995 |  |  |  |  |  |  |  |  | 4 |
| Burgoon 1963 |  |  |  |  |  |  |  |  | 4 |
| Canarozzo 2016 |  |  |  |  |  |  |  |  | 6 |
| Casals 2009 |  |  |  |  |  |  |  |  | 4 |
| Cianchini 2001 |  |  |  |  |  |  |  |  | 5 |
| Cohen 1976 |  |  |  |  |  |  |  |  | 6 |
| Cohen 2003 |  |  |  |  |  |  |  |  | 5 |
| DePanfilis 1981 |  |  |  |  |  |  |  |  | 4 |
| Dellon 1977 |  |  |  |  |  |  |  |  | 6 |
| Dicken 1982 |  |  |  |  |  |  |  |  | 5 |
| Dogan 2011 |  |  |  |  |  |  |  |  | 3 |
| Ellis 1984 |  |  |  |  |  |  |  |  | 5 |
| Exadaktylou 2003 |  |  |  |  |  |  |  |  | 5 |
| Farb 1980 |  |  |  |  |  |  |  |  | 6 |
| Fisher 1955 |  |  |  |  |  |  |  |  | 6 |
| Fishman 1975 |  |  |  |  |  |  |  |  | 4 |
| Flores-Terry 2017 |  |  |  |  |  |  |  |  | 4 |
| Goh 2005 |  |  |  |  |  |  |  |  | 4 |
| Gupta 1990 |  |  |  |  |  |  |  |  | 3 |
| Hesbacher 1970 |  |  |  |  |  |  |  |  | 5 |
| Hussain 2009 |  |  |  |  |  |  |  |  | 4 |
| Itin 2002 |  |  |  |  |  |  |  |  | 5 |
| Ji 2018 |  |  |  |  |  |  |  |  | 4 |
| Katta 2000 |  |  |  |  |  |  |  |  | 3 |
| Katz 2010 |  |  |  |  |  |  |  |  | 4 |
| Kim 2007 |  |  |  |  |  |  |  |  | 6 |
| Kittridge 2010 |  |  |  |  |  |  |  |  | 4 |
| Knulst 1995 |  |  |  |  |  |  |  |  | 4 |
| Krakowski 2015 |  |  |  |  |  |  |  |  | 5 |
| Legrand 2020 |  |  |  |  |  |  |  |  | 4 |
| Letule 2013 |  |  |  |  |  |  |  |  | 5 |
| Leung 2018 |  |  |  |  |  |  |  |  | 5 |
| Liang 2018 |  |  |  |  |  |  |  |  | 5 |
| Macmanus 2001 |  |  |  |  |  |  |  |  | 4 |
| Magdaleno-Tapial 2019 |  |  |  |  |  |  |  |  | 4 |
| Mandel 1979 |  |  |  |  |  |  |  |  | 6 |
| McElroy 1990 |  |  |  |  |  |  |  |  | 5 |
| McKenna 1999 |  |  |  |  |  |  |  |  | 4 |
| Medeiros 2015 |  |  |  |  |  |  |  |  | 4 |
| Mei 2000 |  |  |  |  |  |  |  |  | 3 |
| Meziane 2008 |  |  |  |  |  |  |  |  | 3 |
| Micali 1999 |  |  |  |  |  |  |  |  | 4 |
| Milavec-Puretic 2001 |  |  |  |  |  |  |  |  | 4 |
| Millan-Parrilla 2014 |  |  |  |  |  |  |  |  | 4 |
| O’Malley 1997 |  |  |  |  |  |  |  |  | 3 |
| Oi-Yee Li 2018 |  |  |  |  |  |  |  |  | 4 |
| Oostenbrink 1996 |  |  |  |  |  |  |  |  | 4 |
| Orihuela 1995 |  |  |  |  |  |  |  |  | 4 |
| Oster-Schmidt 1999 |  |  |  |  |  |  |  |  | 4 |
| Palacios-Alvarez 2017 |  |  |  |  |  |  |  |  | 4 |
| Parlak 2001 |  |  |  |  |  |  |  |  | 4 |
| Parslew 1994 |  |  |  |  |  |  |  |  | 2 |
| Peck 1976 |  |  |  |  |  |  |  |  | 5 |
| Peck 1978 |  |  |  |  |  |  |  |  | 5 |
| Perez-Carmona 2011 |  |  |  |  |  |  |  |  | 4 |
| Pettit 2018 |  |  |  |  |  |  |  |  | 3 |
| Pezzini 2015 |  |  |  |  |  |  |  |  | 2 |
| Podgornii 2013 |  |  |  |  |  |  |  |  | 6 |
| Raszewska-Famielic 2015 |  |  |  |  |  |  |  |  | 5 |
| Rodriguez 2018 |  |  |  |  |  |  |  |  | 6 |
| Roos 2008 |  |  |  |  |  |  |  |  | 6 |
| Rubegni 2006 |  |  |  |  |  |  |  |  | 4 |
| Sanchez-Salas 2011 |  |  |  |  |  |  |  |  | 2 |
| Santos-Alarcon 2016 |  |  |  |  |  |  |  |  | 4 |
| Schmidt 2008 |  |  |  |  |  |  |  |  | 5 |
| Schwartz 2011 |  |  |  |  |  |  |  |  | 3 |
| Sfecci 2015 |  |  |  |  |  |  |  |  | 4 |
| Shahidullah 1994 |  |  |  |  |  |  |  |  | 4 |
| Shreberk-Hassidim 2016 |  |  |  |  |  |  |  |  | 4 |
| Sondhi 2020 |  |  |  |  |  |  |  |  | 4 |
| Sprowson 2004 |  |  |  |  |  |  |  |  | 5 |
| Stewart 2008 |  |  |  |  |  |  |  |  | 4 |
| Stuttgen 1977 |  |  |  |  |  |  |  |  | 2 |
| Thomas 1982 |  |  |  |  |  |  |  |  | 4 |
| Thomas 1989 |  |  |  |  |  |  |  |  | 4 |
| Toombs 1989 |  |  |  |  |  |  |  |  | 5 |
| Tsiogka 2015 |  |  |  |  |  |  |  |  | 5 |
| vanDooren-Greebe 1989 |  |  |  |  |  |  |  |  | 6 |
| van'tWesteinde 2006 |  |  |  |  |  |  |  |  | 4 |
| Vender 2016 |  |  |  |  |  |  |  |  | 4 |
| Vieira 2020 |  |  |  |  |  |  |  |  | 4 |
| vonKockritz 2018 |  |  |  |  |  |  |  |  | 4 |
| Wheeland 1985 |  |  |  |  |  |  |  |  | 5 |
| Yoon 2006 |  |  |  |  |  |  |  |  | 5 |
| Zachariae 1979 |  |  |  |  |  |  |  |  | 5 |
| Zamiri 2013 |  |  |  |  |  |  |  |  | 5 |
| Zavattaro 2014 |  |  |  |  |  |  |  |  | 6 |
| Zhang 2008 |  |  |  |  |  |  |  |  | 5 |

**Supplementary Table 2: Risk Of Bias In Non-Randomized Studies - of Interventions (ROBINS-I)**

| Study | Bias due to confounding | Bias in selection of participants into the study | Bias in classification of interventions | Bias due to deviations from intended interventions | Bias due to missing data | Bias in measurement of outcomes | Bias in selection of the reported result | Total Score out of 7 |
| --- | --- | --- | --- | --- | --- | --- | --- | --- |
| Abe 2011 |  |  |  |  |  |  |  | 6 |
| Blackman 1980 |  |  |  |  |  |  |  | 4 |
| Cipollaro 1979 |  |  |  |  |  |  |  | 6 |
| Fulton 1968 |  |  |  |  |  |  |  | 6 |
| Kragballe 1995 |  |  |  |  |  |  |  | 6 |
| Steijlen 1991 |  |  |  |  |  |  |  | 6 |
| Steijlen 1993 |  |  |  |  |  |  |  | 5 |

**Supplementary Table 3: Oral Retinoids for Darier’s Disease**

| **Therapeutic Modality** | **Special Indications** | **Regimen when known** | **Duration of Tx when known (Range)** | **Adverse Events** | **Basis of evidence** | **Grade of Reccomendation** | **Outcomes** |
| --- | --- | --- | --- | --- | --- | --- | --- |
| Oral retinoids | n/a | 0.2-0.5 mg/kg q.d | 2-287 months | 62/88 had mild adverse events, 21/88 experienced severe adverse event. 20/88 patients for which data was available terminated medication due to major adverse events, many others discontinued drug due to minor adverse events which became intolerable, 7/88 discontinued due to improving skin disease ; bone hyperostosis 1/18 | Retrospective non-comparative cohorts ^1,2^ | 2B | +/++/+++ |
| Oral acitretin | Pediatric DD; cornifying DD; hypertrophic DD variant with condyloma-like lesions; acral hemorrhagic DD; pruriginous DDl; DD with Cutis Verticis Gyrata | 0.8-1 mg/kg or 10-60mg q.d  (maintenance 0.3mg/kg or 12.5mg q.d) | 3-22 months or ongoing | aridity cheilitis, increasing fragility of skin, dry mouth and skin, anorexia, pruritus, dry eye, hyperorexia, hearing handicap, brittle nail in one finger, acral hemorrhagic lesions, mild elevation of serum triglyceride levels | Case reports, series, retrospective non-comparative  cohort, non-controlled trials^3,4,13–15,5–12^ | 2A | +/++/+++ |
| Oral isotretinoin  (13-cis-retionic acid) | DD of the scalp causing pityriasis amiantacea | 10-320 mg q.d or 40mg b.i.d or 0.5-7.4mg/kg q.d | 1-55 weeks, intermittent courses (4 months) or ongoing | Epistaxis, cheilitis, xerosis, patient discountinued drug due to side effects (side effects persisting with any dose), (increased triglyceride levels in very high doses), hyperostosis (after 6 months to 7 years), ; conjunctivitis, facial dermatitis, rhinitis sicca with nosebleed, skin fragility, itching, headache, appetite changes, fingertip peeling, inflammation of the urethral meatus, hair thinning, dryness of mouth (with feeling of thirst), allergic reaction, elevated AST,ALT, ALP, ESR | Case reports, non-controlled trials^16,17,26,18–25^ | 2A | +/++/+++ |
| Oral alitretinoin | DD resistant to conventional therapies; DD in women of childbearing age | 30 mg q.d (maintenance 30mg q3days) | 1-18 months | Mild eye dryness, moderate reversible hair loss, headache, elevated total cholesterol, and low-density lipoprotein levels | Case reports^27–30^ | 2B | +++ |
| Systemic Vitamin A | n/a | 25,000-1,000, 000 IU q.d (intramuscular injections of vitamin A for first 4 months) | 14 days-Ongoing (treatment regimen changed at 4 months) | Dry/scaling lips, dry nose, dry skin, headaches, pruritis,sleepiness, drowsiness, desquamation | Case series and report, non-controlled trial ^31–33^ | 2A | +/++/+++ |
| Oral Vitamin A Acid (tretinoin) | n/a | 100mg first 7 days, reduce by 20mg after  (maintenance 20mg b.i.d) | Unknown | Cheilitis, rhinitis sicca, skin cleavage | Case series^34^ | 2B | +/++/+++ |
| Oral Vitamin A palmitate | n/a | 25,000 IU b.i.d, maintenance 25,000 q.d with use of bland emollients in face/skin | Ongoing | None noted | Case report^35^ | 2B | +++ |

**Supplementary Table 4: Other Systemic Treatments for Darier’s Disease**

| **Therapeutic Modality** | **Special Indications** | **Regimen when known** | **Duration of Tx when known (Range)** | **Adverse Events** | **Basis of evidence** | **Grade of Reccomendation** | **Outcomes** |
| --- | --- | --- | --- | --- | --- | --- | --- |
| Methylprednisone | Vesiculo-bullous DD | 0.2 mg/kg qd | Ongoing | None noted | Case report^36^ | 2B | +++ |
| Intravenous immunoglobulin | DD resistant to conventional therapies | 0.4 g/kg infusion q3weeks | Ongoing | None noted | Case report^37^ | 2B | +++ |
| Ciclosporin | Vulval DD; eczematisation of DD | 3-6mg/kg qd | Ongoing or 2-6 months – however relapse occurred after discontinuation | Significant hypertension, renal failure (resulting in discontinuation) | Case report ^38–40^ | 2B | +/++/+++ |
| Oral Magnesium | Pediatric (15yo) DD; DD resistant to conventional therapy | 300mg qd | 1 month | Unpleasant taste | Case report ^41^ | 2B | +++ |
| Oral Penicillamine | n/a | 1200-1500mg q.d (maintenance therapy 900mg q.d) | 2-4 months | Urticaria, eczema, temporary decrease in granulocytes | Case series^42^ | 2B | -/+/++/+++ |
| Systemic antibiotics | n/a | Doxycycline 100 mg q.d $\pm$emollients | 4 weeks | None noted | Case reports^43,44^ | 2B | +++ |
| Oral Contraceptives | Female patients with DD flares during menstruation | Microgynon 50 qd (levonogestrol 125 μg, ethinylestradiol 50 μg) | Ongoing (3 weeks per month) | Weight gain | Case report^45^ | 2B | +/++ |

+: partial improvement, ++: moderate improvement, +++: significant clinical improvement or complete remission

**Supplementary Table 5: Topical Treatments for Darier’s Disease**

| **Therapeutic Modality** | **Special Indications** | **Regimen** | **Duration of Tx** | **Associated Side effects/ adverse Events** | **Basis of evidence** | **Grade of Recommendation** | **Outcome** |
| --- | --- | --- | --- | --- | --- | --- | --- |
| Topical tretinoin | Linear (zosteriform) DD; acral hemorrhagic DD; DD resistant to conventional therapy | 0.05%-0.2% cold cream application q.d/b.i.d;  48hr application of 1% Vitamin A acid under occlusion (plastic wrap) PRN or weekly-quarterly | 1 month-2 years or PRN | Itching, tenderness, skin irritation (resulting in discontinuation) inflammation, desquamation, and severe erythema and burning (resulting in discontinuation) | Case reports, retrospective non-comparative cohort, controlled trial^1,8,53,13,46–52^ | 2A | +/++/+++ |
| Topical isotretinoin  (13-cis-retinoic acid) | Linear (zosteriform) DD | 0.05-0.1% q.a.d, working up to q.d | 1-3 months, ongoing maintenance | burning, erythema, irritation, itching, erythema, tenderness | Case report, non-controlled trial,  Controlled trials^51,54–56^ | 2A | -/+/++/+++ |
| Adapalene Gel | Pediatric DD; acral DD; linear (zosteriform) DD | 0.1% gel qd (+ one week of keratolytic topical therapy for lesions on soles | 3 months/ ongoing | None noted | Case report, controlled trial^57–59^ | 2A | ++/+++ |
| Tazarotene | DD resistant to conventional therapies; pediatric DD | 0.01-0.1% gel q.d. | Two weeks | None noted | Case reports^60,61^ | 2B | +++ |
| Calcipotriol | n/a | 50 μg/g q.d. (less than 120 g/wek) | 6 weeks | Lesional-perilesional skin irritation, worsening of DD (7/12 withdrew from trial due to adverse events, one withdrew voluntarily) | Randomized control trial^62^ | 2A | -/+/++ |
| Topical Tacalcitol Lotion with sunscreen | Localized DD | unknown | Unknown | None noted | Case report, controlled trial^58,63^ | 2A | +/+++ |
| Topical fluocinonide | Acute, eruptive DD in mature adult | Lidex cream 4-8 times q.d | Unknown (at least 9 months) | None noted | Case report^64^ | 2B | ++/+++ |
| Topical tacrolimus | DD in seborrheic areas of torso and face | Initial: 0.1% ointment b.i.d (6 weeks) Maintenance: 0.03% b.i.d for four weeks, then 0.03% qd | 6 weeks (maintenance therapy ongoing) | None noted | Case report^65^ | 2B | +++ |
| Topical pimecrolimus | DD resistant to conventional therapies | 1% cream b.i.d, (maintenance therapy 1% cream two times per week) | 4 weeks (ongoing maintenance) | None noted | Case report^66^ | 2B | +++ |
| Diclofenac Sodium Gel | DD resistant to conventional therapies | 3% gel q.d-b.i.d, 1% b.i.d in patient with suspected DD, but no mutation, clearance in one month | 3-8 months | Skin irritation | Case reports^67–69^ | 2B | ++/+++ |
| Topical 5-flououracil | DD resistant to conventional therapies | 1% 5-FU cream q.d/q.a.d $\pm$acitretin | 2-4 weeks or until clearance | Hyperpigmentation | Case reports, non-controlled trial^70–72^ | 2A | -/++/+++ |

+: partial improvement, ++: moderate improvement, +++: significant clinical improvement or complete remission

**Supplementary Table 6: Procedural Interventions for Darier’s Disease**

| **Surgical Technique** | **Special Indications** | **Regimen** | **Duration of Tx** | **Adverse Events** | **Basis of evidence** | **Grade of Recommendation** | **Comment** |
| --- | --- | --- | --- | --- | --- | --- | --- |
| Surgical excision | Hypertrophic DD; intertriginous or  perianal DD;  DD resistant to conventional therapies; nail thickening in DD | Partial thickness or full-thickness skin/nail (epidermis and dermis) excision | Once | Necrosis and exudate from wound, wound dehiscence, infection, scar formation, hypopigmentation | Case reports^4,73–78^ | 2B | ++/+++ |
|  | Inframammary DD plaques | Bilateral reduction mammaplasty | Once | Necrosis of right nipple-areolar complex | Case report^79^ | 2B | ++ |
| Dermabrasion | n/a | Unknown | Once | unknown | Case series^80^ | 2B | ++ |
| CO_2_ laser | Pediatric segmental DD | 1-12 passes with CO_2_ laser | 1-3 times (6 weeks apart) | irritation and erythema (may be prolonged), transient erythema and edema | Case series, case report^81–85^ | 2B | ++/+++ |
| Erbium-doped yttrium aluminum  Laser (Er:YAG) | Linear (zosteriform) DD | 1-8 passes using an erbium-doped yttrium aluminum laser | 1-4 treatments | Mild hypopigmentation including atrophic hypopigmented spots, temporary pain, erythema and edema | Case report and series^86,87^ | 2B | ++/+++ |
| Flashlamp-Pumped Pulsed-Dye Laser | DD resistant to conventional therapy | Flashlamp-pumped pulsed-dye laser | 1-2 treatments (8 weeks apart) | Purpura with mild crusting, HSV infection flare | Case series and report^88,89^ | 2B | +++ |
| Near-infrared  1450-nm wavelength diode laser | DD reoccurrence after treatment with CO_2_ laser | Near-infrared 1450-nm wavelength diode laser pulses 3-10 times | One course | None noted | Case report^84^ | 2B | +++ |
| Grenz Ray | DD resistant to conventional therapy | Total dose 1,200r | 6 treatments (1-2 weeks apart) | Pain, temporary exacerbation of DD | Controlled trial^90^ | 2A | -/+++ |
| X-ray | DD resistant to conventional therapy | Total dose 450r | 6 treatments (1-2 weeks apart) | Temporary exacerbation of DD | Controlled trial^90^ | 2A | -/+++ |
| Electron Beam radiation therapy | DD resistant to conventional therapies | Total dose of 900 cGy (2500 cGy planed but not tolerated) to 40 Gy | One course | Temporary local dermatitis and moist desquamation, severe dermatitis, striae in treatment areas, mild disease flare outside the treatment area, skin pain, nausea, and vomiting (patient experienced prolonged hospitalization, admission to the intensive care unit), sclerosis and ulcerations | Case series and report^91,92^ | 2B | ++/+++ |
| Photon Radiation therapy | Linear (zosteriform) DD; DD with thickened hyperkeratotic contours; DD resistant to conventional therapies | Total dose 30Gy | One course | Pain | Case series^92^ | 2B | +++ |
| Radiotherapy | DD resistant to conventional therapy | Total dose 40-50 Gy of radiotherapy for treatment of malignancy | One course | Temporary local dermatitis, mild exacerbation of DD | Case report ^93–95^ | 2B | ++/+++ |
| Photodynamic therapy | DD resistant to conventional therapies;  localized plaques of DD | Photodynamic therapy with a light source emitting a spectrum of 420-740 nm for 8-16 minutes  5-ALA  hydrochloride 20% w ⁄ w in Unguentum Merck (Craw-  ford Pharmaceuticals, Milton Keynes, U.K.) and occlu-  ded for four hours under a dressing impermeable to  light. The site to be treated was then examined with a  Wood’s lamp to detect ﬂuorescence of locally synthes-  ized PpIX. Irradiation was subsequently performed with  an incoherent light source (PDT1200 lamp; Wald-  mann, Villingen-Schwenningen, Germany), which  emits ﬁltered light (580–740 nm) at ﬂuence rates of  110–150 mW cm  )2  using a dose of 150 J cm  )2  (range  of exposure times: 16 min 40 s to 22 min 44 s | 1-2 times (2 weeks apart) | hyperkeratotic conﬂuent papules ﬁssures, redness, and edema in previously  uninvolved skin, temporary inflammatory reaction lasting two to three weeks (erythema, exudation and discomfort) | Case report and controlled trial ^96–98^ | 2A | +/++/+++ |

+: partial improvement, ++: moderate improvement, +++: significant clinical improvement or complete remission

**Supplementary Table 7: Combination Treatments for Darier’s Disease**

| Combination Treatment | Special Indications | Duration of Treatment when known | Adverse Events | Basis of evidence | Grade of Recommendation | Outcomes |
| --- | --- | --- | --- | --- | --- | --- |
| Oral acitretin 10-40mg q.d, topical antiseptics and keratolytics b.i.d | Comedonal DD; septic  DD | 6 months- 1 year | None noted | Case reports^99,100^ | 2B | ++/+++ |
| Oral acitretin 50 mg q.d and systemic steroids | n/a | Ongoing | None noted; partial improvement in between periods of severe exacerbation and functional decline resulting in referral to palliative care and death | Case report^101^ | 2B | + |
| Oral isotretinoin 20mg q.d, antiseptic washes with chlorhexidine gluconate, fusidic acid 2% cream, ketoconazole 2% cream and emollients | -Late onset DD | 2 months | None noted | Case report^102^ | 2B | ++/+++ |
| Oral isotretinoin 40mg b.i.d (starting with q.d) and salicylic acid 5% in petrolatum | DD with palmoplantar keratoderma | Ongoing | None noted | Case report^20^ | 2B | ++ |
| Maintenance treatment with topical tretinoin, and oral isotretinoin 20mg q.d during severe flares | Severe DD flares | Ongoing during severe flares | None noted | Case report^8^ | 2B | + |
| Vitamin A 200,000 units and cortisone 150 mg q.d | DD of the larynx | 20 days | None noted | Case report^103^ | 2B | +++ |
| Vitamin A 100, 000 IU and vitamin E 1,600 IU q.d | DD resistant to conventional therapies | Ongoing | None noted | Case report^31^ | 2B | ++/+++ |
| Magnesium 200mg and naltrexone 5mg q.d | n/a | Ongoing | Worsening of DD, itchiness, pain | Case series^104^ | 2B | -/+++ |
| Magnesium 200mg, naltrexone 5mg, and oral retinoids (acitretin 10 mg and isotretinoin 30 mg or acitretin 25 mg)  q.d | n/a | Ongoing | Worsening of DD, itchiness, pain | Case series^104^ | 2B | -/+++ |
| Systemic and topical cycles of antibiotics and antihistamines | Vegetating DD with pseudoepitheliomatous features | n/a | None noted | Case report^105^ | 2B | ++ |
| Vitamin A acid cream and betamethasone valerate | Linear (zosteriform) DD | 2 years | None noted | Case report^47^ | 2B | +++ |
| Tretinoin 0.1% cream and lactic acid 12% in lachydrin lotion b.i.d | Linear (zosteriform) DD | 3 months | Mild erythema | Case report^106^ | 2B | +++ |
| Tretinoin 0.05% or group IV steroid cream (chest)/group V steroid cream (face) on alternating days | n/a | 3 months | None noted | Case report^107^ | 2B | ++ |
| Light curettage followed by photodynamic therapy and tretinoin cream 0.05% q.d | n/a | One course of photodynamic therapy, 3-month topical tretinoin | None noted | Case report^108^ | 2B | -/+/+++ |
| Adapalene gel 0.3% associated with urea 20% and salicylic acid 8% | Segmental DD | 3 months | None noted | Case report^109^ | 2B | +++ |
| Oral antihistamines, 5-fluorouracil 1% cream and topical tazarotene | Bullous-hemorrhagic DD | unknown | None noted | Case report^110^ | 2B | + |
| CO_2_ laser with maintenance using weekly application of tacrolimus 0.1% ointment, tazarotene 0.1% gel, and urea 35% foam | Pediatric DD | 3 laser treatments (2 months apart) topical treatment ongoing | None noted | Case report^83^ | 2B | +++ |
| Complete surgical excision of lesions followed by CO_2_ laser ablation | Condyloma-like DD | One course | None noted | Case report^111^ | 2B | +++ |
| Split-thickness surgical excision followed by dermabrasion | Cornifying DD | Once | Cellulitis, full-thickness skin loss | Case series and report^112,113^ | 2B | ++/+++ |
| Electron beam radiation therapy (total dose 20Gy) and intensity modulated radiation therapy | n/a | One course | None noted | Case report^94^ | 2B | +++ |
| Electron beam radiation and brachytherapy | DD resistant to electron beam and intensity modulated radiation therapy | One course | None noted | Case report^94^ | 2B | +++ |

+: partial improvement, ++: moderate improvement, +++: significant clinical improvement or complete remission

**Supplementary Figure 1: PRISMA Flow Diagram**

Full-text articles assessed for eligibility
(n = 155)

Records excluded
(n = 319)

Records screened
(n = 474)

Records after duplicates removed
(n = 474)

Additional records identified through other sources
(n =0)

Records identified through database searching
(n =474)

## **Identification**

## **Screening**

Full-text articles excluded, with reasons (n = 42)

Full text not available (n=15)

Wrong outcomes (n=15)

Drug discontinued (n=4)

Wrong study design (n=2)

Wrong intervention (n=2)

Duplicate (n=2)

Wrong patient population (n=1)

Wrong Indication (n=1)

## **Eligibility**

Studies discussing treatments of Darier’s disease
(n = 113)

## **Included**

From: Moher D, Liberati A, Tetzlaff J, Altman DG, The PRISMA Group (2009). Preferred Reporting Items for Systematic

Reviews and MetaAnalyses: The PRISMA Statement. PLoS Med 6(7): e1000097. doi:10.1371/journal.pmed1000097

For more information, visit [www.prisma-statement.org](http://www.prisma-statement.org).

**REFERENCES**

1. Burge SM, Wilkinson JD. Darier-White disease: A review of the clinical features in 163 patients. J Am Acad Dermatol. 1992 Jul 1;27(1):40–50.

2. Vieira ML, de Paula Samorano L, da Matta Rivitti-Machado MC, de Oliveira ZNP. Darier disease: long-term treatment with systemic retinoids at a tertiary hospital [Internet]. Vol. 18, JDDG - Journal of the German Society of Dermatology. Wiley-VCH Verlag; 2020 [cited 2020 Jul 13]. p. 628–30. Available from: https://onlinelibrary.wiley.com/doi/abs/10.1111/ddg.14113

3. Zhang X, Luo Q, Li C, He Y, Xu X. Clinical investigation of acitretin in children with severe inherited keratinization disorders in China. J Dermatolog Treat [Internet]. 2008 Jan 12 [cited 2020 Jul 8];19(4):221–8. Available from: http://www.tandfonline.com/doi/full/10.1080/09546630801955150

4. Sprowson AP, Jeffery SLA, Black MJM. Darier’s disease, an unusual problem and solution. J Hand Surg Am. 2004 Jun 1;29 B(3):293–5.

5. Katta R, Reed J, Wolf JE. Cornifying Darier’s disease. Int J Dermatol [Internet]. 2000 Nov [cited 2020 Jul 9];39(11):844–5. Available from: http://doi.wiley.com/10.1046/j.1365-4362.2000.00994.x

6. Borgogna C, Zavattaro E, Dell’Oste V, Mondini M, Valente G, Colombo E, et al. No indications for HPV involvement in the hypertrophic skin lesions of a Darier disease case without *ATP2A2* gene mutations. J Cutan Pathol [Internet]. 2009 Sep [cited 2020 Jul 10];36(9):1005–9. Available from: http://doi.wiley.com/10.1111/j.1600-0560.2009.01182.x

7. Zavattaro E, Celasco M, Delrosso G, Ferri S, Bornacina C, Valente G, et al. Acitretin-induced acral hemorrhagic lesions in Darier-White disease. Cutis [Internet]. 2014 Dec [cited 2020 Jul 11];94(6):E1-5. Available from: http://www.ncbi.nlm.nih.gov/pubmed/25566576

8. Flores-Terry MÁ, García-Arpa M, Llamas-Velasco M, Mendoza-Chaparro C, Ramos-Rodríguez C, De Darier E. Acral Hemorrhagic Darier Disease . Vol. 108, Actas Dermosifiliogr. 2017.

9. Liang J, Chen P, Chen H, Tian X, Wu Z, Zhang S, et al. Long-term safety and efficacy of continuous acitretin monotherapy for three children with different severe hyperkeratotic disorders in China. J Dermatol [Internet]. 2018 Aug 1 [cited 2020 Jul 12];45(8):1003–8. Available from: http://doi.wiley.com/10.1111/1346-8138.14462

10. Blanchet-Bardon C, Nazzaro V, Rognin C, Geiger JM, Puissant A. Acitretin in the treatment of severe disorders of keratinization: Results of an open study. J Am Acad Dermatol. 1991 Jun 1;24(6):982–6.

11. Magdaleno‐Tapial J, Valenzuela‐Oñate C, Martínez‐Domenech Á, García‐Legaz‐Martínez M, Sánchez‐Carazo JL, Alegre‐de Miquel V. A pruriginous eruption on the back, worsening in the summer. Clin Exp Dermatol [Internet]. 2019 Oct 21 [cited 2020 Jul 13];44(7):794–6. Available from: https://onlinelibrary.wiley.com/doi/abs/10.1111/ced.13865

12. DOOREN-GREEBE RJ, KERKHOF PCM, HAPPLE R. Acitretin monotherapy in Darier’s disease. Br J Dermatol [Internet]. 1989 Sep [cited 2020 Jul 13];121(3):375–9. Available from: http://doi.wiley.com/10.1111/j.1365-2133.1989.tb01432.x

13. Goh BK, Ang P, Goh CL. Darier’s disease in Singapore. Br J Dermatol [Internet]. 2005 Feb [cited 2020 Jul 14];152(2):284–8. Available from: http://doi.wiley.com/10.1111/j.1365-2133.2004.06364.x

14. Itin PH, Happle R. Darier Disease with Paired Segmental Manifestation of Either Excessive or Absent Involvement: A Further Step in the Concept of Twin Spotting. Dermatology [Internet]. 2002 [cited 2020 Jul 9];205(4):344–7. Available from: https://www.karger.com/Article/FullText/66423

15. Parlak M, Erdem T, Karakuzu A, Güleç AI, Akdeniz N. Darier’s disease seen with cutis verticis gyrata . Vol. 81, Acta Dermato-Venereologica. 2001. p. 75.

16. Bhat R, Ullal K, Pinto A, Sukumar D. Darier-White disease in siblings responding to isotretinoin. Indian Dermatol Online J [Internet]. 2010 [cited 2020 Jul 12];1(1):18. Available from: http://www.idoj.in/text.asp?2010/1/1/18/73252

17. Ellis CN, Madison KC, Pennes DR, Martel W, Voorhees JJ. Isotretinoin therapy is associated with early skeletal radiographic changes. J Am Acad Dermatol [Internet]. 1984 Jan [cited 2020 Jul 13];10(6):1024–9. Available from: https://linkinghub.elsevier.com/retrieve/pii/S0190962284803291

18. Dicken CH, Bauer EA, Hazen PG, Krueger GG, Marks JG, McGuire JS, et al. Isotretinoin treatment of Darier’s disease. J Am Acad Dermatol. 1982 Apr 1;6(4):721–6.

19. ARCHER CB, ELIAS PM, LOWE NJ, GRIFFITHS WAD. Extensive spinal hyperostosis in a patient receiving isotretinoin-progression after 4 years of etretinate therapy. Clin Exp Dermatol [Internet]. 1989 Jul [cited 2020 Jul 14];14(4):319–21. Available from: http://doi.wiley.com/10.1111/j.1365-2230.1989.tb01993.x

20. Kim C, journal WF-D online, 2007 undefined. Keratosis follicularis (Darier-White disease), with an unusual palmoplantar keratoderma. pubmed.ncbi.nlm.nih.gov [Internet]. [cited 2020 Jul 10]; Available from: https://pubmed.ncbi.nlm.nih.gov/17511940/

21. Peck GL, Yoder FW, Olsen TG, Pandya MD, Butkus D. Treatment of darier’s disease, lamellar ichthyosis, pityriasis rubra pilaris, cystic acne, and basal cell carcinoma with oral 13-cis-retinoic acid. Dermatology [Internet]. 1978 [cited 2020 Jul 12];157(Supplement 01):11–2. Available from: https://pubmed.ncbi.nlm.nih.gov/150981/

22. Peck GL, Yoder FW. TREATMENT OF LAMELLAR ICHTHYOSIS AND OTHER KERATINISING DERMATOSES WITH AN ORAL SYNTHETIC RETINOID. Lancet. 1976 Nov 27;308(7996):1172–4.

23. Farb RM, Lazarus GS, Chiaramonti A, Goldsmith LA, Gilgor RS, Balakrishnan C V. The effect of 13-cis retinoic acid on epidermal lysosomal hydrolase activity in Darier’s disease and pityriasis rubra pilaris. J Invest Dermatol [Internet]. 1980 Aug [cited 2020 Jul 14];75(2):133–5. Available from: https://linkinghub.elsevier.com/retrieve/pii/S0022202X15458695

24. Orihuela E, Tyring SK, Pow-Sang M, Dozier S, Cirelli R, Arany I, et al. Development of Human Papillomavirus Type 16 Associated Squamous Cell Carcinoma of the Scrotum in a Patient with Darier’s Disease Treated with Systemic Isotretinoin. J Urol. 1995 Jun 1;153(6):1940–3.

25. Blackman HJ, Rodrigues MM, Peck GL. Corneal Epithelial Lesions in Keratosis Follicularis (Darier’s Disease). Ophthalmology [Internet]. 1980 [cited 2020 Sep 27];87(9):931–43. Available from: https://pubmed-ncbi-nlm-nih-gov.proxy.bib.uottawa.ca/7413157/

26. Hussain W, Coulson IH, Salman WD. Pityriasis Amiantacea as the sole manifestation of Darier’s disease. Clin Exp Dermatol [Internet]. 2009 Jun [cited 2020 Sep 27];34(4):554–6. Available from: https://pubmed-ncbi-nlm-nih-gov.proxy.bib.uottawa.ca/19522991/

27. Letulé V, Herzinger T, Ruzicka T, Molin S. Treatment of Darier disease with oral alitretinoin. Clin Exp Dermatol [Internet]. 2013 Jul [cited 2020 Jul 8];38(5):523–5. Available from: https://pubmed.ncbi.nlm.nih.gov/23777494/

28. Shreberk-Hassidim R, Sheffer S, Horev L, Zlotogorski A, Ramot Y. Successful treatment of refractory Darier disease with alitretinoin with a follow up of over a year: a case report. Dermatol Ther [Internet]. 2016 Jul 1 [cited 2020 Jul 11];29(4):222–3. Available from: http://doi.wiley.com/10.1111/dth.12366

29. Anuset D, Goutorbe C, Bernard P, Reguiai Z. Efficacy of oral alitretinoin for the treatment of Darier disease: A case report. J Am Acad Dermatol [Internet]. 2014 Aug [cited 2020 Jul 13];71(2):e46–8. Available from: https://linkinghub.elsevier.com/retrieve/pii/S019096221401007X

30. Zamiri M, Munro CS. Successful treatment with oral alitretinoin in women of childbearing potential with Darier’s disease [Internet]. Vol. 169, British Journal of Dermatology. Br J Dermatol; 2013 [cited 2020 Jul 17]. p. 709–10. Available from: https://pubmed.ncbi.nlm.nih.gov/23551220/

31. Ayres S. Darier’s Disease: Update on an Effective New Therapy [Internet]. Vol. 119, Archives of Dermatology. American Medical Association; 1983 [cited 2020 Jul 13]. p. 710. Available from: https://jamanetwork-com.proxy.bib.uottawa.ca/journals/jamadermatology/fullarticle/544396

32. Thomas JR, Cooke JP, Winkelmann RK. High-Dose Vitamin A Therapy for Darier’s Disease. Arch Dermatol [Internet]. 1982 Nov 1 [cited 2020 Jul 14];118(11):891–4. Available from: https://jamanetwork-com.proxy.bib.uottawa.ca/journals/jamadermatology/fullarticle/543663

33. Burgoon CF, Graham JH, Urbach F, Musgnug R. Effect of Vitamin A on Epithelial Cells of Skin: The Use of Vitamin A in the Treatment of Diseases Characterized by Abnormal Keratinization. Arch Dermatol [Internet]. 1963 [cited 2020 Jul 18];87(1):63–80. Available from: https://pubmed.ncbi.nlm.nih.gov/14017007/

34. STÜTTGEN G, IPPEN H, MAHRLE G. ORAL VITAMIN A ACID IN TREATMENT OF DERMATOSES WITH PATHOLOGIC KERATINIZATION [Internet]. Vol. 16, International Journal of Dermatology. Int J Dermatol; 1977 [cited 2020 Jul 7]. p. 500–2. Available from: https://pubmed.ncbi.nlm.nih.gov/142750/

35. Sondhi M, Vashist S, Mahajan VK. Darier’s Disease - Response to Oral Vitamin A: Report of a Case and Brief Review. Indian Dermatol Online J [Internet]. 2020 [cited 2020 Jul 13];11(1):72–8. Available from: /pmc/articles/PMC7001418/?report=abstract

36. Mei S, Amato L, Gallerani I, Perrella E, Caproni M, Palleschi GM, et al. A case of vesiculo-bullous Darier’s disease associated with bipolar psychiatric disorder. J Dermatol [Internet]. 2000 [cited 2020 Jul 9];27(10):673–6. Available from: https://pubmed.ncbi.nlm.nih.gov/11092274/

37. Legrand A, Darrigade A-S, Taieb A, Milpied B, Seneschal J. Response to low-dose intravenous immunoglobulin in a case of recalcitrant Darier disease. JAAD Case Reports [Internet]. 2020 [cited 2020 Jul 13];6(3):189. Available from: https://www.ncbi.nlm.nih.gov/pmc/articles/PMC7033304/

38. Stewart LC, Yell J. Vulval Darier’s disease treated successfully with ciclosporin. J Obstet Gynaecol (Lahore) [Internet]. 2008 Jan 2 [cited 2020 Jul 8];28(1):108–9. Available from: http://www.tandfonline.com/doi/full/10.1080/01443610701844077

39. Gupta AK, Ellis CN, Nickoloff BJ, Goldfarb MT, Ho VC, Rocher LL, et al. Oral Cyclosporine in the Treatment of Inflammatory and Noninflammatory Dermatoses: A Clinical and Immunopathologic Analysis. Arch Dermatol [Internet]. 1990 Mar 1 [cited 2020 Jul 10];126(3):339–50. Available from: https://jamanetwork.com/

40. SHAHIDULLAH H, HUMPHREYS F, BEVERIDGE GW. Darier’s disease: severe eczematization successfully treated with cyclosporin. Br J Dermatol [Internet]. 1994 [cited 2020 Jul 13];131(5):713–6. Available from: https://pubmed.ncbi.nlm.nih.gov/7999607/

41. Oi-Yee Li H, Colantonio S, Kanigsberg N. Treatment of Darier’s disease with oral magnesium: a case report. SAGE Open Med Case Reports [Internet]. 2018 Jan [cited 2020 Jul 7];6:2050313X1879507. Available from: /pmc/articles/PMC6134490/?report=abstract

42. Beer WE, Lyle WH. Penicillamine for the treatment of Darier’s disease and other disorders of keratin formation. Lancet. 1966;288(7477):1337–40.

43. Safa G, Sfecci A, Orion C, Darrieux L, Tisseau L. Extensive Darier Disease Successfully Treated with Doxycycline Monotherapy. Case Rep Dermatol [Internet]. 2015 [cited 2020 Jul 11];7:311–5. Available from: www.karger.com/cde

44. Pettit C, Ulman CA, Spohn G, Kaffenberger J. A case of segmental darier disease treated with doxycycline monotherapy. Dermatol Online J [Internet]. 2018 Mar 1 [cited 2020 Jul 11];24(3). Available from: https://europepmc.org/article/med/29634885

45. OOSTENHRINK JH, COIIEN EB, STEIJLEN PM, KERKHOF PCM. Oral contraceptives in the treatment of Darier-White disease- a case report and review of the literature. Clin Exp Dermatol [Internet]. 1996 Nov [cited 2020 Jul 9];21(6):442–4. Available from: http://doi.wiley.com/10.1111/j.1365-2230.1996.tb00152.x

46. Meziane M, Chraibi R, Kihel N, Hassam B, Senouci K. Linear Darier disease. Dermatol Online J [Internet]. 2008 Dec 15 [cited 2020 Jul 8];14(12):11. Available from: http://www.ncbi.nlm.nih.gov/pubmed/19265624

47. O’Malley MP. Localized Darier Disease. Arch Dermatol [Internet]. 1997 Sep 1 [cited 2020 Jul 9];133(9):1134. Available from: http://archderm.jamanetwork.com/article.aspx?doi=10.1001/archderm.1997.03890450084010

48. Dogan S, Karaduman A, Erkin G, Gokoz O. Effective treatment of linear Darier’s disease with topical retinoids: Case report and review of the literature. Vol. 19, Acta Dermatovenerologica Croatica. 2011. p. 206–9.

49. Vender R, Vender R. Acral Hemorrhagic Darier’s Disease: A Case Report. J Cutan Med Surg [Internet]. 2016 Sep 7 [cited 2020 Jul 11];20(5):478–80. Available from: http://www.ncbi.nlm.nih.gov/pubmed/26992421

50. Panfilis G, Manara GC, Ferrari C, Tedeschi F, Allegro F. Darier’s Keratosis Follicularis: An Ultrastructural Study During and After Topical Treatment with Retinoic Acid Alone or in Combination with 5-Fluorouracil*. J Cutan Pathol [Internet]. 1981 Jun 1 [cited 2020 Jul 14];8(3):214–8. Available from: http://doi.wiley.com/10.1111/j.1600-0560.1981.tb01000.x

51. Steijlen PM, Happle R, Van Muijen GNP, Van De Kerkhof PCM. Topical treatment with 13-cis-retinoic acid improves darier’s disease and induces the expression of a unique keratin pattern. Dermatology [Internet]. 1991 [cited 2020 Jul 10];183(3):178–83. Available from: https://pubmed.ncbi.nlm.nih.gov/1715297/

52. Hesbacher EN. Zosteriform Keratosis Follicularis Treated Topically With Tretinoin. Arch Dermatol [Internet]. 1970 Aug 1 [cited 2020 Jul 7];102(2):209–12. Available from: https://jamanetwork-com.proxy.bib.uottawa.ca/journals/jamadermatology/fullarticle/531735

53. Fulton JE, Gross PR, Cornelius CE, Kligman AM. Darier’s Disease: Treatment With Topical Vitamin A Acid. Arch Dermatol [Internet]. 1968 Oct 1 [cited 2020 Jul 7];98(4):396–9. Available from: https://jamanetwork.com/

54. Steijlen PM, Reifenschweiler DOH, Ramaekers FCS, van Muijen GNP, Happle R, Link M, et al. Topical treatment of ichthyoses and Darier’s disease with 13-cis-retinoic acid - A clinical and immunohistochemical study. Arch Dermatol Res [Internet]. 1993 Jun [cited 2020 Jul 10];285(4):221–6. Available from: https://pubmed.ncbi.nlm.nih.gov/7688204/

55. Burge SM, Buxton PK. Topical isotretinoin in Darier’s disease. Br J Dermatol [Internet]. 1995 [cited 2020 Jul 9];133(6):924–8. Available from: https://pubmed.ncbi.nlm.nih.gov/8547046/

56. KE M, MY W, D B. Treatment of Unilateral Darier’s Disease With Topical Isotretinoin. Clin Exp Dermatol [Internet]. 1999 [cited 2020 Jul 9];24(5). Available from: https://pubmed.ncbi.nlm.nih.gov/10564339/

57. Cianchini, L. Colonna, D. Camaioni, G. Acral Darier’s Disease Successfully Treated with Adapalene. Acta Derm Venereol [Internet]. 2001 Jan 1 [cited 2020 Jul 9];81(1):57–8. Available from: https://pubmed.ncbi.nlm.nih.gov/11411920/

58. Abe M, Inoue C, Yokoyama Y, Ishikawa O. Successful treatment of Darier’s disease with adapalene gel. Pediatr Dermatol [Internet]. 2011 Mar [cited 2020 Jul 12];28(2):197–8. Available from: http://doi.wiley.com/10.1111/j.1525-1470.2009.01077.x

59. Casals M, Campoy A, Aspiolea F, Carrasco M, Camps A. Successful treatment of linear Darier’s disease with topical adapalene. J Eur Acad Dermatology Venereol [Internet]. 2009 Feb [cited 2020 Jul 13];23(2):237–8. Available from: http://doi.wiley.com/10.1111/j.1468-3083.2008.02815.x

60. Oster-Schmidt. The treatment of Darier’s disease with topical tazarotene. Br J Dermatol [Internet]. 1999 Sep [cited 2020 Jul 9];141(3):603–4. Available from: http://doi.wiley.com/10.1046/j.1365-2133.1999.03089.x

61. Micali G, Nasca MR. Tazarotene gel in childhood Darier disease. Pediatr Dermatol [Internet]. 1999 May 1 [cited 2020 Jul 9];16(3):243–4. Available from: http://www.ncbi.nlm.nih.gov/pubmed/10383788

62. Kragballe K, Steijlen PM, Ibsen HH, Van De Kerkhof PCM, Esmann J, Sorensen LH, et al. Efficacy, Tolerability, and Safety of Calcipotriol Ointment in Disorders of Keratinization: Results of a Randomized, Double-blind, Vehicle-Controlled, Right/Left Comparative Study. Arch Dermatol [Internet]. 1995 May 1 [cited 2020 Jul 10];131(5):556–60. Available from: https://jamanetwork-com.proxy.bib.uottawa.ca/journals/jamadermatology/fullarticle/556640

63. Abe M, Yasuda M, Yokoyama Y, Ishikawa O. Successful treatment of combination therapy with tacalcitol lotion associated with sunscreen for localized Darier’s disease. J Dermatol [Internet]. 2010 Aug [cited 2020 Jul 12];37(8):718–21. Available from: https://pubmed.ncbi.nlm.nih.gov/20649714/

64. Fishman HC. Acute, Eruptive Darier Disease (Keratosis Follicularis): Occurrence in an Adult. Arch Dermatol [Internet]. 1975 Feb 1 [cited 2020 Jul 14];111(2):221–2. Available from: http://archderm.jamanetwork.com/article.aspx?doi=10.1001/archderm.1975.01630140079009

65. Rubegni P, Poggiali S, Sbano P, Risulo M, Fimiani M. A case of Darier’s disease successfully treated with topical tacrolimus. J Eur Acad Dermatology Venereol [Internet]. 2006 Jan [cited 2020 Jul 10];20(1):84–7. Available from: https://pubmed.ncbi.nlm.nih.gov/16405615/

66. PÉREZ-CARMONA L, FLETA-ASÍN B, MORENO-GARCÍA-DEL-REAL C, JAÉN-OLASOLO P. Successful treatment of Darier’s disease with topical pimecrolimus. Eur J Dermatology [Internet]. 2011 Mar [cited 2020 Jul 11];21(2):301–2. Available from: http://www.john-libbey-eurotext.fr/medline.md?doi=10.1684/ejd.2011.1309

67. Millán-Parrilla F, Rodrigo-Nicolás B, Molés-Poveda P, Armengot-Carbó M, Quecedo-Estébanez E, Gimeno-Carpio E. Improvement of Darier disease with diclofenac sodium 3% gel. J Am Acad Dermatol [Internet]. 2014 [cited 2020 Jul 7];70(4). Available from: https://pubmed.ncbi.nlm.nih.gov/24629373/

68. Palacios-Álvarez I, Andrés-Ramos I, Silva MY, Simal G. Treatment of Darier’s disease with diclofenac sodium 3% gel. Dermatol Ther [Internet]. 2017 May 1 [cited 2020 Jul 11];30(3):e12478. Available from: http://doi.wiley.com/10.1111/dth.12478

69. Santos-Alarcon S, Sanchis-Sanchez C, Mateu-Puchades A. Diclofenac sodium 3% gel for darier’s disease treatment. Dermatol Online J [Internet]. 2016 Apr 18 [cited 2020 Jul 11];22(4). Available from: http://www.ncbi.nlm.nih.gov/pubmed/27617470

70. KNULST AC, FAILLE HB, VLOTEN WA. Topical 5-fluorouracil in the treatment of Darier’s disease. Br J Dermatol [Internet]. 1995 Sep [cited 2020 Jul 8];133(3):463–6. Available from: http://doi.wiley.com/10.1111/j.1365-2133.1995.tb02679.x

71. Yoon TY, Kim JW, Kim MK. Successful treatment of Darier disease with topical 5-fluorouracil [8]. Vol. 154, British Journal of Dermatology. 2006. p. 1210–2.

72. Schmidt H, Ochsendorf FR, Wolter M, Geisslinger G, Ludwig RJ, Kaufmann R. Topical 5-fluorouracil in Darier disease [Internet]. Vol. 158, British Journal of Dermatology. Br J Dermatol; 2008 [cited 2020 Jul 10]. p. 1393–6. Available from: https://pubmed.ncbi.nlm.nih.gov/18410420/

73. Ji WB, Joung S-Y, Min B-W, Um JW. Surgical excision for non-familial hypertrophic Darier’s disease. ANZ J Surg [Internet]. 2018 Jan 1 [cited 2020 Jul 11];88(1–2):E77–8. Available from: http://doi.wiley.com/10.1111/ans.13255

74. Lee Dellon A, Chretien PB, Peck GL. Successful treatment of darter’s disease by partial-thickness removal of skin. Plast Reconstr Surg [Internet]. 1977 [cited 2020 Jul 11];59(6):823–30. Available from: https://pubmed.ncbi.nlm.nih.gov/323892/

75. Ahcan U, Dolenc-Voljc M, Zivec K, Zorman P, Jurcic V. The surgical treatment of hypertrophic intertriginous Darier’s disease. J Plast Reconstr Aesthetic Surg [Internet]. 2009 Nov [cited 2020 Jul 12];62(11):e442–6. Available from: https://linkinghub.elsevier.com/retrieve/pii/S1748681508008358

76. TOOMBS EL, PECK GL. Electrosurgical Treatment of Etretinate‐Resistant Darier’s Disease. J Dermatol Surg Oncol [Internet]. 1989 Dec [cited 2020 Jul 13];15(12):1277–80. Available from: http://doi.wiley.com/10.1111/j.1524-4725.1989.tb03147.x

77. WHEELAND RG, GILMORE WA. The Surgical Treatment of Hypertrophic Darier’s Disease. J Dermatol Surg Oncol [Internet]. 1985 Apr 1 [cited 2020 Jul 13];11(4):420–3. Available from: https://onlinelibrary-wiley-com.proxy.bib.uottawa.ca/doi/full/10.1111/j.1524-4725.1985.tb01294.x

78. Baran R. An effective surgical treatment for nail thickening in Darier’s disease. J Eur Acad Dermatology Venereol [Internet]. 2005 Nov [cited 2020 Jul 13];19(6):689–91. Available from: http://doi.wiley.com/10.1111/j.1468-3083.2005.01263.x

79. Cohen PR. Darier disease: sustained improvement following reduction mammaplasty. Cutis [Internet]. 2003 Aug 1 [cited 2020 Jul 9];72(2):124–6. Available from: http://www.ncbi.nlm.nih.gov/pubmed/12953935

80. Zachariae H. Dermabrasion of Hailey-Hailey disease and Darier’s disease. Vol. 27, Journal of the American Academy of Dermatology.

81. McElroy JA, Mehregan DA, Roenigk RK. Carbon dioxide laser vaporization of recalcitrant symptomatic plaques of Hailey-Hailey disease and Darier’s disease. J Am Acad Dermatol. 1990 Nov 1;23(5):893–7.

82. Raszewska-Famielec M, Dudra-Jastrzębska M, Borzęcki A, Chodorowskaf G. Darier-White disease treated with fractional CO2 laser in two cases. Dermatol Ther [Internet]. 2015 Jul 1 [cited 2020 Jul 11];28(4):254–7. Available from: http://doi.wiley.com/10.1111/dth.12226

83. Krakowski AC, Nguyen TA, Eichenfield LF. Treatment of segmental keratosis follicularis (darier disease) using ablative fractional laser resurfacing. Vol. 41, Dermatologic Surgery. Lippincott Williams and Wilkins; 2015. p. 516–8.

84. Brown VL, Kelly SE, Burge SM, Walker NPJ. Extensive recalcitrant Darier disease successfully treated with laser ablation [Internet]. Vol. 162, British Journal of Dermatology. Br J Dermatol; 2010 [cited 2020 Jul 13]. p. 227–9. Available from: https://pubmed.ncbi.nlm.nih.gov/19912213/

85. Benmously R, Litaiem N, Hammami H, Badri T, Fenniche S. Significant alleviation of Darier’s disease with fractional CO2 laser. J Cosmet Laser Ther [Internet]. 2015 Mar 4 [cited 2020 Jul 21];17(2):77–9. Available from: http://www.tandfonline.com/doi/full/10.3109/14764172.2014.988728

86. Beier C, Kaufmann R. Efficacy of erbium:YAG laser ablation in Darier disease and Hailey- Hailey disease. Arch Dermatol [Internet]. 1999 Apr 1 [cited 2020 Jul 9];135(4):423–7. Available from: https://jamanetwork.com/

87. KATZ TM, FIROZ BF, GOLDBERG LH, FRIEDMAN PM. Treatment of Darierʼs Disease Using a 1,550-nm Erbium-Doped Fiber Laser. Dermatologic Surg [Internet]. 2010 Jan [cited 2020 Jul 10];36(1):142–6. Available from: http://journals.lww.com/00042728-201001000-00025

88. Roos S, Karsai S, Ockenfel HM, Raulin C. Successful treatment of darier disease with the flashlamp-pumped pulsed-dye laser [Internet]. Vol. 144, Archives of Dermatology. American Medical Association; 2008 [cited 2020 Jul 8]. p. 1073–5. Available from: https://jamanetwork.com/

89. Cannarozzo G, Bonciani D, Sannino M, Tamburi F, Morini C, Piccolo D, et al. Dye Laser Treatment for Darier Disease: Results of a Case Series [Internet]. Vol. 34, Photomedicine and Laser Surgery. Mary Ann Liebert Inc.; 2016 [cited 2020 Jul 11]. p. 305–7. Available from: https://www.liebertpub.com/doi/10.1089/pho.2015.4034

90. CIPOLLARO VA, SHAPS R. THE TREATMENT OF DARIER’S DISEASE. Int J Dermatol [Internet]. 1979 Sep 1 [cited 2020 Jul 14];18(7):580–3. Available from: http://doi.wiley.com/10.1111/j.1365-4362.1979.tb01977.x

91. Kittridge A, Wahlgren C, Fuhrer R, Zirwas M, Patton T. Treatment of recalcitrant Darier’s disease with electron beam therapy. Dermatol Ther [Internet]. 2010 May 1 [cited 2020 Jul 10];23(3):302–4. Available from: http://doi.wiley.com/10.1111/j.1529-8019.2010.01327.x

92. Leung N, Cardones AR, Larrier N. Long-term improvement of recalcitrant Darier disease with photon and electron beam radiation therapy. JAAD Case Reports [Internet]. 2018 Nov 1 [cited 2020 Jul 27];4(10):1062–4. Available from: https://www.ncbi.nlm.nih.gov/pmc/articles/PMC6250900/

93. Podgornii A, Ciammella P, Ramundo D, Iotti C. Efficacy of the Radiotherapy on Darier’s Disease: An Indirect Evidence. Case Rep Dermatol Med [Internet]. 2013 [cited 2020 Jul 11];2013. Available from: http://dx.

94. Rodriguez LM, Kazemi T, Cheng CE, Kang JJ, Beron PJ, Kozma BD, et al. Focal multimodality radiation therapy: A promising treatment for recalcitrant Darier disease. Dermatol Ther [Internet]. 2018 Jul 1 [cited 2020 Jul 14];31(4):e12641. Available from: http://doi.wiley.com/10.1111/dth.12641

95. Manus MP Mac, Cavalleri G, Ball DL, Beasley M, Rotstein H, Mckay MJ. Exacerbation, then Clearance, of Mutation-Proven Darier’s Disease of the Skin after Radiotherapy for Bronchial Carcinoma: A Case of Radiation-Induced Epidermal Differentiation?

96. van’t Westeinde S, Sanders C, van Weelden H. Photodynamic therapy in a patient with Darier’s disease. J Eur Acad Dermatology Venereol [Internet]. 2006 Apr 21 [cited 2020 Jul 8];0(0):060606032107043-??? Available from: http://doi.wiley.com/10.1111/j.1468-3083.2006.01548.x

97. Exadaktylou D, Kurwa HA, Calonje E, Barlow RJ. Treatment of Darier’s disease with photodynamic therapy. Br J Dermatol [Internet]. 2003 Sep 1 [cited 2020 Jul 9];149(3):606–10. Available from: https://pubmed.ncbi.nlm.nih.gov/14510996/

98. Avery HL, Hughes BR, Coley C, Cooper HL. Clinical improvement in Darier’s disease with photodynamic therapy. Australas J Dermatol [Internet]. 2010 Feb [cited 2020 Jul 12];51(1):32–5. Available from: http://doi.wiley.com/10.1111/j.1440-0960.2009.00589.x

99. von Köckritz A, Rütten A, Böhm M, Metze D. Treatment-recalcitrant comedones. JDDG J der Dtsch Dermatologischen Gesellschaft [Internet]. 2018 Apr 1 [cited 2020 Jul 7];16(4):494–6. Available from: http://doi.wiley.com/10.1111/ddg.13374

100. Milavec-Puretić V, Lipozenčić J, Šustić N, Pećina-Šlaus N. Sepsis as an unusual event in dyskeratosis follicularis. undefined. 2001;

101. Ashok Kumar P, Paulraj S, Dutta S. Debilitating Darier’s Disease and Its Impact on the Quality of Life. Cureus. 2020 May 15;12(5).

102. Beiu C, Giurcaneanu C, Mihai M, Popa LG, Hage R. Darier Disease – A Clinical Illustration of Its High Variable Expressivity. Cureus [Internet]. 2019 Dec 5 [cited 2020 Jul 11];11(12). Available from: /pmc/articles/PMC6942508/?report=abstract

103. Fisher ER, Kyler SL. Darier’s disease of the larynx. AMA Arch Otolaryngol [Internet]. 1955 Oct 1 [cited 2020 Jul 19];62(4):438–41. Available from: https://jamanetwork.com/

104. Boehmer D, Eyerich K, Darsow U, Biedermann T, Zink A. Variable response to low-dose naltrexone in patients with Darier disease: a case series. J Eur Acad Dermatology Venereol [Internet]. 2019 May 1 [cited 2020 Jul 13];33(5):950–3. Available from: https://onlinelibrary.wiley.com/doi/abs/10.1111/jdv.15457

105. Pezzini C, Vassallo C, Grasso V, Rivetti N, Borroni G. Pseudoepitheliomatous Changes in a Case of Vegetating Darier–White Disease. Am J Dermatopathol [Internet]. 2015 Apr 7 [cited 2020 Jul 7];37(4):323–5. Available from: http://journals.lww.com/00000372-201504000-00013

106. Thomas I, Shockman J, David Epstein J. Linear keratosis follicularis: A specific entity? Report of a case responding to combined topical retinoid and α-hydroxy acid therapy. J Am Acad Dermatol [Internet]. 1989 [cited 2020 Jul 26];20(6):1122–3. Available from: https://pubmed.ncbi.nlm.nih.gov/2754061/

107. Schwartz JL, Clinton TS. Darier’s Disease Misdiagnosed as Severe Seborrheic Dermatitis. Mil Med. 2011 Dec;176(12):1457–9.

108. Amerio P, Gobello T, Mazzanti C, Giaculli E, Ruggeri S, Sordi D, et al. Photodynamic therapy plus topical retinoids in Darier’s disease. Photodiagnosis Photodyn Ther. 2007 Mar 1;4(1):36–8.

109. Medeiros PM, Alves NR de M, Trujillo JM, da Silva CC, de Faria PCP, da Silva RS. Segmental Darier’s disease: A presentation of difficult diagnosis. An Bras Dermatol [Internet]. 2015 Jun 1 [cited 2020 Jul 11];90(3):S62–5. Available from: /pmc/articles/PMC4540510/?report=abstract

110. Sánchez-Salas M, Latasa de Aranibar F, Oncíns Torres R, Gambó Grasa P. Bullous-hemorrhagic Darier disease. Skinmed [Internet]. 2011 [cited 2020 Jul 11];9(1):65–6. Available from: https://europepmc.org/article/med/21409967

111. Tsiogka A, Stückler C, Prodinger C, Koller J. Condyloma-like Darier’s disease of the inguinal region: resolution after surgical excision and CO2 laser ablation. JDDG J der Dtsch Dermatologischen Gesellschaft [Internet]. 2015 Nov 1 [cited 2020 Jul 11];13(11):1180–4. Available from: http://doi.wiley.com/10.1111/ddg.12831

112. Mandel MA. Cornifying Darier’s disease. Plast Reconstr Surg [Internet]. 1979 Feb [cited 2020 Jul 11];63(2):167–72. Available from: http://www.ncbi.nlm.nih.gov/pubmed/419195

113. Cohen IK, Kraemer KH, Peck GL. Cornifying Darier Disease— A Unique Variant: II. Surgical Treatment. Arch Dermatol [Internet]. 1976 Apr 1 [cited 2020 Jul 13];112(4):504–6. Available from: https://jamanetwork.com/
